# Self-Reported Fatigue and Associated Factors Six Years after Stroke

**DOI:** 10.1371/journal.pone.0161942

**Published:** 2016-08-30

**Authors:** Marie Elf, Gunilla Eriksson, Sverker Johansson, Lena von Koch, Charlotte Ytterberg

**Affiliations:** 1 Department of Nursing, School of Education, Health and Social Studies, Dalarna University, Falun, Sweden; 2 Department of Neurobiology, Care Sciences and Society, Karolinska Institutet, Huddinge, Stockholm, Sweden; 3 Function Area Occupational Therapy and Physiotherapy, Karolinska University Hospital, Stockholm, Sweden; 4 Department of Neurology, Karolinska University Hospital, Stockholm, Sweden; Banner Alzheimer's Institute, UNITED STATES

## Abstract

Several studies have found that fatigue is one of the most commonly reported symptoms after stroke and the most difficult to cope with. The present study aimed to investigate the presence and severity of self-reported fatigue six years after stroke onset and associated factors. The cohort “Life After Stroke Phase I” (n = 349 persons) was invited at six years to report fatigue (Fatigue Severity Scale 7-item version), perceived impact of stroke and global recovery after stroke (Stroke Impact Scale), anxiety and depression (Hospital Anxiety and Depression Scale), life satisfaction (Life Satisfaction Checklist) and participation in everyday social activities (Frenchay Activities Index). At six years 37% of the 102 participants in this cross-sectional study reported fatigue. The results showed that in nearly all SIS domains the odds for post-stroke fatigue were higher in persons with a higher perceived impact. Furthermore, the odds for post-stroke fatigue were higher in those who had experienced a moderate/severe stroke and had signs of depression and anxiety. Fatigue is still present in one-third of persons as long as six years after stroke onset and is perceived to hinder many aspects of functioning in everyday life. There is an urgent need to develop and evaluate interventions to reduce fatigue.

## Introduction

Several studies have found that post-stroke fatigue (PSF) is one of the most commonly reported symptoms after stroke [1–5] and the most difficult to cope with [2,6,7]. PSF has been described as a subjective feeling consisting of a profound sense of tiredness and a lack of mental and/or physical energy after stroke [1,3,4,8]. PSF occurs even without any demonstrable exertion and can lead to the person’s difficulty or inability in the performance of even routine and minor activities[4,9], thus potentially having an impact on everyday life [7,9–12].

PSF is common immediately after stroke, and although long-term follow-ups are unusual, evidence indicates that PSF can be a long-lasting impairment [8,11,13,14]. The reported prevalence of PSF ranges between 23% and 75%, depending on the sample, the time after stroke and the method of assessing fatigue[3,8,11,15,16]. Studies show that between 40% and 74% report fatigue two years after stroke onset [3,14,15,17] and as many as 58% three years after stroke[18]. Prevalence of fatigue in a general population has been reported to be 11–23% [19,20].

The exact mechanism behind PSF, its pattern and associated factors are still elusive [1,8,12,21]. Patients who do not have PSF initially can develop fatigue later on [7] although PSF in the early phase of stroke is a strong predictor of prolonged PSF [15]. Wu and colleagues [7] propose fatigue as part of an evolving process, i.e. there may be specific factors associated with fatigue at an early stage after stroke and others at a later stage. Early PSF may be triggered by biological factors (severity and location of the lesion), as opposed to later PSF, which may be associated more with psychological (depression and anxiety) and behavioural factors (coping strategies and inactivity) [7].

Studies have shown that PSF is associated not only with reduced quality of life [3,4,18] and poor neurological recovery [13] but also with a higher risk of death [3,14,18]. In addition, results from studies indicate that PSF has an impact on independence and the performance of daily activities and impedes patients’ participation in rehabilitation programs [3,4,22–24] which may thereby impair health and functioning.

In a recent meta-analysis including studies with follow-ups at between two and 18 months after stroke, an association was found between PSF and depressive symptoms, as well as a tendency towards an association between PSF and anxiety [25]. In addition, longitudinal studies have shown that depression and anxiety at baseline are associated with PSF 18 month after stroke onset [24,26].

Although PSF is a commonly reported problem that negatively affects rehabilitation and recovery after stroke, PSF remains a neglected problem [12]. Persons who have had a stroke and health-care professionals repeatedly request information about how to manage and prevent fatigue after stroke [2,6]. However, today there is not yet sufficient evidence of PSF to guide care and treatment [27,28] not least when it comes to addressing PSF with a long-term perspective. In order to develop effective treatments and support for patients with PSF, it is crucial to understand PSF from a long-term perspective and to understand how various factors are associated with PSF [29].

The present study aimed to investigate the presence of self-reported PSF six years after stroke onset and associated factors.

## Materials and Methods

### Participants and procedure

This study is a six-year follow up on the study “Life After Stroke Phase 1” (LAS-1), a prospective cohort study of the rehabilitation process after stroke. A detailed description of the study has been reported elsewhere [2]. Initially, LAS-1 included 349 patients diagnosed with stroke who were admitted to stroke units at Karolinska University Hospital in Stockholm, Sweden, between 2006 and 2007. At baseline, the first week after stroke onset, demographic data were retrieved from the medical records. The Barthel Index (BI)[30] was used to categorise stroke severity. A score of ≤14 was classified as severe, 15–49 as moderate and ≥50 as mild stroke severity [31]. At six years after stroke onset, a letter was sent out to all survivors from LAS-1, containing information about the follow-up study. Informed consent was obtained in connection with the data collection, which was carried out through structured face-to-face interviews in the participants’ homes. The interviews were performed by an occupational therapist or a physiotherapist trained for the purpose. The participants in the present study are persons included in LAS-1 who agreed to participate in a six-year follow-up and who answered a self-report fatigue questionnaire.

### Outcome assessments and instruments

#### Fatigue

Self-reported fatigue (the dependent variable) was assessed by the Fatigue Severity Scale 7-item version (FSS-7) [32], a validated self-report questionnaire that assesses the severity of fatigue and its impact on people’s functioning in everyday life. In the present study, the FSS-7[33] was used as it has been shown to have better psychometric properties than the FSS-9 [34] The final score is the mean of the seven items graded from 1 (strong disagreement) to 7 (strong agreement). In this study, we categorised the presence of fatigue as a mean score of ≥ 4 points, in line with previous studies including patients who have had stroke [33].

#### Impact of stroke

Perceived impact of stroke and global recovery after stroke were assessed using the Stroke Impact Scale (SIS) [35]. The instrument assesses the perceived impact of stroke in eight domains: strength, hand function, activities in daily life, mobility, communication, emotion, memory and thinking, and participation. The total score for each domain is calculated using an algorithm. The domain score ranges from 0 points (the maximum perceived impact of stroke) to 100 points (no impact). In addition, one question regarding the participant’s perception of recovery after stroke is rated on a visual analogue scale (VAS) ranging from 0 (no recovery) to 100 (maximum recovery).

#### Participation in complex and social activities

Participation in everyday social activities was assessed using the Frenchay Activities Index (FAI) [36] which scores the frequency of 15 activities in three domains: domestic tasks (0–15 points), work/leisure (0–18 points) and outdoor activities (0–12 points). The FAI consists of a single summary score ranging from 0 (inactive) to 45 points (very active). In the present study, a score ≥15 was considered as being active, in line with previous studies of people who have had a stroke [37].

#### Life satisfaction

Life satisfaction was assessed by way of the Life Satisfaction Checklist (LiSat-11) [38] an instrument frequently used in people who have had a stroke. In the present study the item on global life satisfaction was used, in which overall life satisfaction is rated on a scale from 1 to 6, where 1 = very dissatisfied and 6 = very satisfied. For the analysis, the scores were dichotomized as satisfied (scores of 5–6) vs not satisfied (scores of 1–4)[39].

#### Anxiety and depression

Signs of anxiety and depression were assessed using the Hospital Anxiety and Depression Scale (HADS), a self-reported questionnaire comprising 14 items [40]. The HADS includes anxiety (HADS-A) and depression (HADS-D) subscales, each comprising seven questions. For each question, the participant was asked to rate his/her emotional state over the previous seven days on a scale of 0 points (no symptoms) to 3 points (maximum impairment). In this study, a cut-off of 4, which has been recommended for persons who have had a stroke [41], was used for HADS-A and HADS-D respectively.

### Statistical analysis

Univariable logistic regression analyses were performed to explore plausible associations of the independent variables with the presence of PSF. Due to the small numbers of persons with moderate (n = 9) and severe (n = 5) stroke those groups were merged into one category, moderate-severe, in all analyses. The SAS® System 9.3, SAS Institute Inc., Cary, NC, USA was used for the statistical analysis.

### Ethics

The study has been conducted according to the principles expressed in the Declaration of Helsinki. Written and verbal informed consent was obtained in connection with the data collection, which was carried out through structured face-to-face interviews in the participants’ homes. The participants' signed written consent was stored in a locked compartment. The study was approved by the Stockholm Regional Ethical Review Board, which also approved the consent procedure.

## Results

Of the 349 persons with stroke included in the LAS-1 study, 121 participated in the six-year follow-up, 166 were deceased, 44 declined participation and 18 could not be reached. Of the 121 participants, 19 had not answered the FSS-7 due to for example cognitive impairment or severe illness, and thus 102 persons were included in the present study. The mean time for follow-up was six years after stroke, ranging from 67 to 89 months. Table 1 shows disease-related and socio-demographic information at baseline of the 102 persons in the present study.

**Table 1:** Disease-related and socio-demographic characteristics at baseline.

	Total sample, n = 102,n (%)
Gender, male/female	57 (56) / 45 (44)
Age, mean years (SD), range	62 (14), 24–85
Civil status, living together / living alone	53 (52) / 49 (48)
Type of stroke, infarction / haemorrhage	88 (86) / 14 (14)
Localization, right / left / both / cerebellum / unclear	40 (39) / 50 (49) / 1 (1) / 8 (8) / 3 (3)
Previous stroke	20 (20)
Previous TIA	5 (5)
Hypertension	50 (49)
Diabetes	17 (17)

At the six-year follow-up, 37% of the participants reported presence of PSF. Results from the univariable logistic regression analyses are presented in Table 2. In nearly all SIS domains the odds for PSF were higher in persons with a higher perceived impact. Furthermore, the odds for PSF were higher in those who had experienced a moderate/severe stroke and had signs of depression and anxiety.

**Table 2:** Characteristics of persons who have had stroke, categorised according to the presence or absence of fatigue and univariable logistic regression for the association between the independent variables and fatigue, odds ratios (OR), 95% confidence intervals (CI) and p values.

Independent variables	Fatigue n = 38	Non-fatigue n = 64	Fatigue Univariate logistic regression OR (95%CI)	P value
Gender, n (%)				
• Men	20 (53)	37 (58)	0.81 (0.36–1.81)	0.61
• Women	18 (47)	27 (42)	1	
Age*, mean (SD)	63 (14)	61 (14)	1.01 (0.98–1.04)	0.57
Stroke severity*, n (%)				
• Moderate/severe	11 (29)	3 (5)	8.28 (2.14–32.10)	0.002
• Mild	27 (71)	61 (95)	1	
Stroke Impact Scale, 0–100, mean (SD)				
• Strength	66 (25)	80 (23)	0.98 (0.96–0.99)	0.01
• Memory/thinking	79 (19)	91 (11)	0.95 (0.92–0.98)	0.001
• Emotion	75 (16)	83 (19)	0.98 (0.95–1.0)	0.04
• Communication	82 (20)	91 (15)	0.97 (0.95–1.00)	0.02
• ADL	79 (25)	87 (18)	0.98 (0.96–1.00)	0.06
• Mobility	77 (22)	87 (19)	0.98 (0.96–1.00)	0.02
• Hand function	69 (30)	81 (31)	0.99 (0.98–1.00)	0.08
• Participation	69 (22)	83 (18)	0.97 (0.95–0.99)	0.002
• Recovery	65 (23)	77 (26)	0.98 (0.97–1.00)	0.02
Frenchay Activities Index, 0–45, median (IQR)	28 (18–35)	31 (23–38)		
• Inactive, <15, n (%)	7 (18)	9 (14)	1.36 (0.46–4.00)	0.582
• Active, ≥15, n (%) Frenchay Activities Index domains, median (IQR)	31 (82)	54 (86)	1	
• Domestic, 0–15	12 (8–15)	13 (9–15)	0.97 (0.89–1.10)	0.53
• Work/leisure, 0–18	12 (7–14)	13 (9–15)	0.94 (0.86–1.02)	0.13
• Outdoor, 0–12	5 (3–7)	6 (4–9)	0.87 (0.77–1.00)	0.05
Hospital Anxiety and Depression Scale				
• Depression subscale, 0–21, median (IQR)	4 (2–7)	1 (0–4)		
• Signs of depression, >4, n (%)	20 (63)	20 (31)	2.44 (1.07–5.59)	0.03
• No signs of depression, ≤4, n (%)	18 (37)	44 (69)	1	
Anxiety subscale, 0–21, median (IQR)	5 (2–7)	2 (0–3)		
• Signs of anxiety, >4, n (%)	22 (58)	15 (24)	4.40 (1.85–10.47)	<0.001
• No signs of anxiety, ≤4, n (%)	16 (42)	48 (76)	1	
Life Satisfaction Checklist, 0–6, median (IQR)	5 (4–5)	5 (4–5)		
• Not satisfied, 1–4, n (%)	4 (39)	26 (32)	0.88 (0.38–2.04)	0.77
• Satisfied, 5–6, n (%)	22 (61)1	36 (58)	1	

*At inclusion

## Discussion

Our study showed that PSF is common six years after stroke onset and is associated with stroke severity, signs of depression and anxiety and a perceived impact of stroke in many areas of functioning. It is well known from previous studies that PSF is common after stroke, but it has not been shown previously that PSF is still present and perceived to hinder one’s daily activities as long as six years after onset.

In our study, the proportion of PSF (37%) is slightly lower than in previous studies conducted up to 36 months after stroke (40–74%) [3, 14, 15, 17]. The variation in the prevalence of PSF may reflect the complexity of PSF and the fact that comparisons are problematic because of the different assessment methods used [12,42]. In addition, the persons included in our study were advanced in age. Younger persons might be more aware of fatigue, because they tend to be more active and have to return to working life after the acute phase [19,43].

We found an association between stroke severity and PSF, which is supported by other studies, which show that a more severe disability, as measured by the BI or the Modified Rankin Scale, is the most important predictor of fatigue [3,10,14,42,44]. This may indicate an association with the severity of the stroke or with persons with greater disability needing more energy to perform daily activities and thus experiencing more fatigue than persons with lesser disability. It has been suggested that persons who have had stroke compensate for physical or cognitive deficits, and that the extra effort results in fatigue [17]. Interestingly, no association was found between PSF and FAI, meaning that persons who experienced PSF did not experience reduced participation in everyday social activities. Earlier studies have shown that because of their unpredictable tiredness, persons with PSF often adapt to their current status by avoiding activities that are important to them; such adaptation includes strategies to avoid meetings with other people [45]. A plausible explanation for our results may be that the persons had adapted to the situation and thus performed the activities presented in the FAI, but we have no information on the participants’ satisfaction with the quality of how the activities were performed. It is also possible that fatigue has an impact on activities not covered by the FAI [46].

In the present study, an association between PSF and signs of anxiety and depression was shown. Our results are in line with the findings of a recent meta-analysis where a significant association between PSF and depressive symptoms was found as well as a tendency towards an association between PSF and anxiety.

Fatigue might be a symptom of depression rather being an independent symptom [1,17]. However, there has been an increase in research indicating that PSF occurs independently from depression [17,45,47,48]. Our results with regard to anxiety concur with those of a previous study in which higher anxiety predicted the presence of fatigue [47]. Consequently, a reduction in a person’s anxiety may also reduce fatigue.

Research into PSF provides a mixed and often conflicting picture, with no clear understanding of what factors are associated with its occurrence. Thus, there are challenges connected to the management of PSF and evidence-based interventions are lacking [27,49]. Psychological interventions such as cognitive behavioural therapy have been shown to reduce fatigue in people with multiple sclerosis [50] cancer [51] and chronic fatigue syndrome [52]. However, studies are needed to evaluate whether similar or other interventions are suitable for PSF.

The clinical implications of our findings include the importance of identifying persons at risk of PSF. Previous studies have shown that PSF is often neglected or not noticed unless specific questions are asked by health-care professionals treating patients with stroke [10]. Eijsden and colleagues[29] have argued that fatigue soon after stroke should be an indicator for follow-ups along the patient’s trajectory of care. PSF is a serious problem which needs better monitoring and management. Thus, it is important for health-care professionals involved in rehabilitation services to pay attention to fatigue when planning and implementing rehabilitation activities. Persons who have had stroke need to be prepared for the possibility of PSF and its challenges and impact on everyday life. Nonetheless, the current results suggest that health-care professionals need to increase their knowledge about how to identify a person’s problem with PSF after stroke.

The main strengths of the study are the long-term follow-up, the fact that all stroke patients admitted to Karolinska University Hospital’s stroke units were eligible to participate, the use of face-to-face interviews for data collection, and the valid and reliable outcome measure.

A limitation is the cross-sectional design, which made it impossible to investigate the relationship between PSF in the acute phase and PSF at the follow-up, as well as causal associations between fatigue and other factors. Moreover, we did not ask what medications the participants were taking at the six-year follow up.

In conclusion, the study shows that PSF is present as long as six years after stroke onset and warrants attention in both clinical practice and research. Particular attention needs to be paid to those who have had severe stroke and those with signs of depression and anxiety. The nature of PSF in the subacute and chronic phases and its contributing factors still need to be scrutinised.

## Supporting Information

S1 Dataset Independent variables, columns A-S: Sex (1 = male, 2 = female), Stroke severity (1 = mild, 2 = moderate/severe), HADS anxiety (0 = no anxiety, 1 = anxiety), HADS depression (0 = no depression, 1 = depression), SIS, column F-N (mean), FAI inactive (0 = inactive, 1 = active), FAI Domains (Domestic, Leisure-Work, Outdoor), columns P-R (sum), LiSat 11 (0 = dissatisfied, 1 = satisfied). Dependent variable, columns T (0 = no fatigue, 1 = fatigue). (DOCX)
