# Supplementary material for: Self-Reported Fatigue and Associated Factors Six Years after Stroke
Source: PLoS One. 2016 Aug 30;11(8):e0161942. doi: 10.1371/journal.pone.0161942 (PMC5004801; doi:10.1371/journal.pone.0161942)
Supplement: S1 Dataset — (DOCX) [file pone.0161942.s001.docx]

**Supporting Information**

S1 Dataset. Independent variables, columns A-S: Sex (1 = male, 2 = female), Stroke severity (1 =mild, 2 = moderate/severe), HADS anxiety (0 = no anxiety, 1 = anxiety), HADS depression (0 = no depression, 1 = depression), SIS, column F-N (mean), FAI inactive (0 = inactive, 1 = active), FAI Domains (Domestic, Leisure-Work, Outdoor), columns P-R (sum), LiSat 11 (0 = dissatisfied, 1 = satisfied). Dependent variable, columns T (0 = no fatigue, 1 = fatigue).
